# Experimental and Computational Study on Conductors Bearing Capacity in Offshore Drilling

**DOI:** 10.1155/2022/2372575

**Published:** 2022-05-27

**Authors:** Nanding Hu, Jin Yang, Bao Suduna, Jiakang Wang, Yida Ding, Xun Liu, Chen Yu

**Affiliations:** ^1^China University of Petroleum-Beijing, Beijing 102249, China; ^2^Tianjin Branch of CNOOC Ltd, Tianjin 300450, China; ^3^Hainan Branch of CNOOC Ltd, Haikou 570100, China

## Abstract

The height of the cementing cement sheath of the conductor for offshore drilling is the key factor affecting wellhead stability. For the influence of the insufficient return height of cement slurry on the mechanical behavior of the conductor after running the conductor by shallow water exploration well drilling, the axial load distribution characteristics and bearing capacity mechanism of conductor were first analyzed from the perspective of operation characteristics of the drilling method. Second, a calculation model of axial bearing capacity of the conductor with the size of the conductor and the return height of cement slurry as variables was established on the basis of the hole enlargement that often occurs during drilling. In addition, the law of the influence of the return height of cement slurry on the ultimate bearing capacity of conductor and the strength difference of the two cementing surfaces between conductor and cement ring and between the cement sheath and submarine soil layer and its changing rules with time were explored and studied by field experiment. It was concluded that the friction between the cement sheath and the soil layer is the key factor that decides the main bearing capacity of the conductor.

## 1. Introduction

The drilling method is one of the main methods of running the conductor in shallow-water drilling and has the advantages of strong adaptability to the shallow seabed soil and great bearing capacity [1–3]. The general step of the drilling method is to drill a wellbore larger than the diameter of the conductor with a bit. The conductor is put into the wellbore and the annulus is filled between the conductor and the wellbore with cement slurry. The cement slurry is consolidated into cement sheath to bond the soil with the conductor to provide lateral friction for the pipe string. However, cement slurry often cannot return to the designed height in real operation due to the difficulty in estimating the amount of lost circulation and cement slurry in the shallow seabed. The bearing capacity provided by the conductor is closely correlated to the height of the cement sheath and the cementing strength of the cement slurry. The insufficient return height of cement slurry causes complicated accidents such as more serious offshore wellhead sinking and even instability [4–6]. Therefore, the study on the influence of cement slurry return height and the setting time on the ultimate bearing capacity of the conductor is of great significance to the construction of shallow water exploration well.

Roy E. Olson and KARLSRUD K theoretically analyzed the axial bearing capacity of steel piles in sand and clay [7, 8]. D. Gouvenot calculated the bearing capacity of cemented piles in a marine environment [9]. Nevertheless, these studies did not focus on the conductor and did not specifically analyze the influence of cement sheath height on the mechanical behavior of the conductor from the perspective of offshore drilling. In real operation, on the premise that the design value of the bearing capacity of the conductor meets the wellhead stability requirements, the sinking of the conductor still occurs, which proves that the factors affecting the actual bearing capacity of the conductor have not yet been fully grasped. As a result, the actual bearing capacity of the conductor cannot be accurately predicted [10].

Therefore, for the operating characteristics of running the conductor by offshore drilling, the axial load distribution characteristics and bearing capacity mechanism of the conductor were first analyzed in this paper from the perspective of operation characteristics of the drilling method. Besides, the influence of hole enlargement on bearing capacity was considered during running the conductor drilling. A calculation model of the ultimate bearing capacity of the conductor considering the return height of the cement sheath was established. Finally, the accuracy of the calculation model of the ultimate bearing capacity was verified by simulation experiments; and the influence of the setting time on the ultimate bearing capacity was explored.

## 2. Ultimate Bearing Capacity Model of Conductors Installed by Drilling

**Figure 1:**
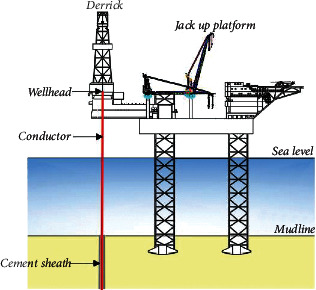
Schematic diagram of conductors in drilling operation.

**Figure 2:**
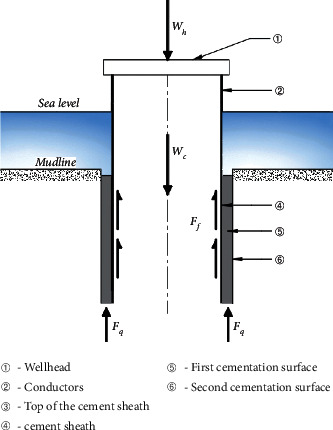
Analysis of axial stress of the conductors run by the method of drilling.

As an important equipment connecting wellhead and seabed in drilling operations, the main function of the conductor is to isolate seawater, provide a closed space for drilling operations, and provide axial bearing capacity for the wellhead (shown in Figure 1). The conductor is generally run by drilling during the construction of shallow water exploration well. The general steps of the drilling method are to drill the conductor to the desired depth with the bit and then to fill the annulus formed between the hole and the conductor with cement slurry. The next step will be carried out after the cement slurry in the annulus is consolidated [11]. The structure diagram of the well for running the conductor by the drilling method is shown in Figure 2.

The load analysis of the conductor showed that the axial force of the conductor is a system that balances the dead load of the conductor and the wellhead load by lateral friction and tip resistance, as shown in Figure 2. The axial bearing capacity provided by the conductor should exclude its own weight. Therefore, the ultimate bearing capacity of the conductor can be expressed as follows:

(1) $$\begin{array}{c} F_{\text{c}}=F_{f}+F_{q}-W_{c}. \end{array}$$

The results showed that the cementing strength of the cement sheath-conductor cemented surface (First cementation surface) is greater than that of the formation-cement sheath cemented surface (second cementation surface) [12, 13]. According to the principle that the weak cemented surface is destroyed first, the second cementation surface is the key factor that decides the ultimate bearing capacity provided by the formation. Assuming that the conductor is in contact with the *n* layer of soil below the mud surface, the friction force on the lateral wall of the conductor is as follows:

(2) $$\begin{array}{c} F_{f}=\sum_{i=1}^{n}F_{i}\\ =\sum_{i=1}^{n-1}f_{i}\pi d_{c}H_{i}+f_{n}\pi d_{c}\left(L-\sum_{i=1}^{n-1}H_{i}\right). \end{array}$$

The tip resistance force of assembly of conductors and cement sheath can be expressed as follows:

(3) $$\begin{array}{c} F_{q}=q_{n}A_{n}. \end{array}$$

The formula for calculating the ultimate bearing capacity of the conductor is obtained by substituting (2) and (3) into (1):

(4) $$\begin{array}{c} F_{\text{c}}=\sum_{i=1}^{n-1}f_{i}\pi d_{c}H_{i}+f_{n}\pi d\left(L-\sum_{i=1}^{n-1}H_{i}\right)+q_{n}A_{n}-m_{c}L. \end{array}$$

According to the strength relation between the two cemented planes, the conductors and the cement sheath should be seen as a whole. Consequently, the value of *d* should be the diameter of a circle formed by the outer diameter of the cement sheath and intersection point of formation, namely, the actual borehole size. But the seawater is utilized as a drilling fluid in the surface drilling, and its density may be less than the collapse pressure of shallow formation sometimes, and consequently causes formation collapse and hole enlargement. Therefore, the actual borehole size cannot be replaced with the bit size. The actual borehole size can be expressed as follows:

(5) $$\begin{array}{c} d=d_{c}\beta . \end{array}$$

Under the condition of the impermeable well wall, the effective stress at the hole collapse can be obtained by calculation according to the linear void elasticity theory. Then, according to the Mohr–Coulomb criterion, the forecast model of borehole enlargement coefficient can be obtained by combining the effective stress at the hole collapse with the ambient stress of well wall [14, 15],

(6) $$\begin{array}{c} \beta =\frac{r_{i}}{r}\\ =\sqrt{\frac{\eta p-2\text{CK}+\alpha p_{p}\left(K^{2}-1\right)-{\text{QK}}^{2}}{\rho_{m}\left(K^{2}+\eta \right)H}\times 100\%}, \end{array}$$

where

(7) $$\begin{array}{c} p=\frac{\sigma_{h1}+\sigma_{h2}}{2}\left(1+\beta^{2}\right)+\frac{\sigma_{h1}-\sigma_{h2}}{2}\left(1+3\beta^{4}\right),\\ Q=\frac{\sigma_{h1}+\sigma_{h2}}{2}\left(1-\beta^{2}\right)-\frac{\sigma_{h1}-\sigma_{h2}}{2}\left(1-4\beta^{2}+3\beta^{4}\right),\\ K=\cot\left(45^{\circ }-\frac{\phi }{2}\right). \end{array}$$

Wellhead load refers to the total load weight borne by the wellhead at the drilling stage and is also an important factor in the design of the conductor's setting depth [16–19]. The wellhead load varies at different construction stages. It is concluded in the combination of actual construction that the wellhead load is the maximum after the technical casing (13–3/8in. casing) is run and before the cement slurry of the technical casing is completely consolidated. Namely,

(8) $$\begin{array}{c} W_{h}=W_{1}+W_{2}. \end{array}$$

## 3. Influence of Cement Return Height

During cementing, the cement slurry is injected from the bottom of the hole through a cementing line into the annulus between the conductor and the soil. The insufficient return height of cement slurry refers to that the return height of cement slurry is lower than the design height (mud surface) due to the infiltration of some cement slurry into the formation during cementing and the decrease of the volume of cement slurry during consolidation. The insufficient return height of cement slurry will result in the failure of the conductor and cement sheath system to cement to the upper soil, losing the lateral friction provided by this part.

**Figure 3:**
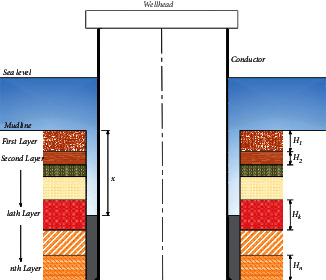
Schematic diagram of insufficient return of drilling cement slurry.

Assuming that the return height of cement slurry is -xm. In other words, the distance between the cement consolidation surface and mud line is xm (as shown in Figure 3). At this time, the residual lateral friction of the conductor can be expressed as follows:

(9) $$\begin{array}{c} F_{c}=f_{k}\pi \beta d_{c}\left(\sum_{i=1}^{k}H_{i}-x\right)+\sum_{i=\text{k}+1}^{n-1}f_{i}\pi \beta d_{c}H_{i}+f_{n}\pi \beta d_{c}\left(L-\sum_{i=1}^{n-1}H_{i}\right)+q_{n}A_{n}-m_{c}L. \end{array}$$

In real operation, the return height of cement slurry is ensured to reach or get close to the design height by setting the injection rate of the cement slurry to 2–3 times the annulus volume [20]. In circumstances where there is sufficient volume of cement slurry, its return height is only affected by the dehydration and consolidation volume shrinkage of cement slurry. Assuming that the volume shrinkage of the cement slurry after complete consolidation in the undersea environment is *λ*. Therefore, the shortage height of cement slurry after complete consolidation can be expressed as follows:

(10) $$\begin{array}{c} x=\left(\frac{\lambda }{\lambda -1}\right)L. \end{array}$$

In addition to the return height of cement slurry, the cementing strength of the second cementation surface is also one of the key factors affecting the ultimate bearing capacity of the conductor[21]. The cement slurry commonly used in offshore drilling is Class *G* cement, which consolidates in a high moisture content environment to provide support for subsequent operations. Experiments have proved that when the conventional cementing method is adopted and the curing time is 7 d under the curing condition of 45 °C, the cementing strength of the second cementation surface is 0.277 MPa, which is 218.4% higher than the 0.087 MPa when the setting time is 2 days[22, 23]. Therefore, the setting time affects the cementing strength of the second cementation surface and then influences the ultimate bearing capacity of the conductor.

## 4. Field Simulation Experiment

In order to explore and study the relationship between the ultimate bearing capacity of the conductor and the return height of cement sheath and the setting time, a simulation experiment of the ultimate bearing capacity of the conductor was designed and carried out.

### 4.1. Variables

According to (9), the return height of the cement slurry is the key factor for the ultimate bearing capacity of the conductor. Therefore, seven different return heights of cement sheath, 0 m, -1 m, -1.5 m, -2 m, -2.5 m, -3 m, and -3.5 m, were selected as test variables. The returned height of 0 m indicates that the cement slurry returns to the mud surface height. Other values mean that the cement slurry returns to a height below the mud surface. Second, when the return height of cement slurry is 0 m, eight different setting times, 0.5 days, 1 days, 2 days, 3 days, 5 days, 7 days, 14 days, and 28 days, were selected to test the ultimate bearing capacity.

At present, there is no definite standard or specification for the determination of the ultimate bearing capacity of the conductor. Hence, in this paper, the actual drilling operation conditions are considered in accordance with the standard for steel pipe pile in the “Code for Pile Foundation of Harbor Engineering” to determine whether the conductor reaches the ultimate bearing capacity [24, 25].

### 4.2. Experimental System and Implementation Process

**Figure 4:**
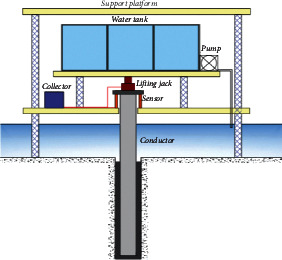
Simulation experiment design.

**Figure 5:**
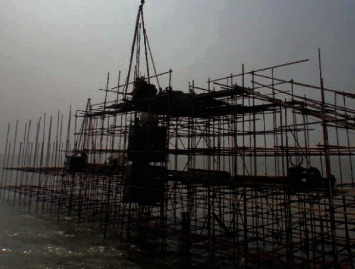
Experiment field of cementing.

The diameter of the bit is 142.9 mm. The experimental system includes a conductor (the diameter is 114.3 mm), drilling equipment, cementing equipment, loading water tank, Jack, water pump, support platform, displacement sensor, load sensor, and data collector. The test system for the ultimate bearing capacity of the conductor is shown in Figure 4 and 5.

First of all, the location of the cementing experiment is identified and soil samples were collected and analyzed. The parameters of the soil within the experimental site are listed in Table 1. Several holes with a depth of 10.2 m and a diameter of 142.9 mm were drilled at the preset experimental sites. The prefabricated conductors were run into these holes. In order to avoid any interference between different models, the distance between two adjacent holes should be at least five times the diameter of holes [26–28]. Second, the cementing equipment was connected; and the cement slurry was injected from the bottom in the annulus between the conductor and the hole. The return height of cement was controlled by the precalculated injection rate. Finally, wellhead load was applied to the conductor with different setting times and different return heights and the settlement of wellhead was detected until reaching its limit value.

### 4.3. Result Analysis

**Figure 6:**
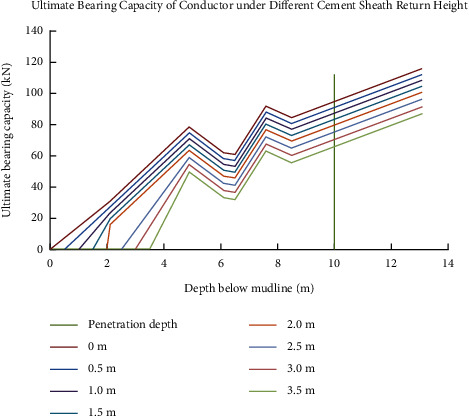
Ultimate bearing capacity of conductors under different cement sheath return heights.

According to (9) and parameters of soil within the experimental site, the changing curves of the ultimate bearing capacity of the conductor model under different conductor's setting depth are shown in Figure 6.

Load-displacement curves at different return heights of cement sheath are also shown in the figure below. In order to ensure the accuracy of the experimental data, the same experiment was carried out three times and averaged to calculate the error between the experimental value of the ultimate bearing capacity and the calculated value of the model (Table 2).

The experimental results showed that the ultimate bearing capacity of the conductor decreased linearly with the decrease of the return height of the cement slurry. When the return height of the cement sheath is 3.5 m below the mud surface, the ultimate bearing capacity reached 30.61%. The error between the ultimate bearing capacity of the conductor obtained by the simulation experiment and the calculated value of (9) is less than 5%, indicating that the accuracy of the calculation model is greater than 95%.

The ultimate bearing capacity of the conductor under different setting times is listed in Table 3. The experiment was performed three times under the same setting times, ensuring the accuracy of experimental data.

**Figure 7:**
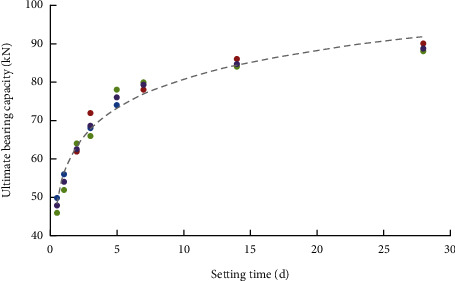
Ultimate bearing capacity varies with setting times.

The experimental results showed that the ultimate bearing capacity of the conductor increased first and then tended to be stable with the increase of the setting time(Figure 7). When the setting time is 0.5 days, the average ultimate bearing capacity is 48 kN, which is 51.2% of the maximum ultimate bearing capacity; when the setting time is 2 days, the average value of the ultimate bearing capacity increases to 63.67 kN, which is 67.9% of the maximum ultimate bearing capacity; when the setting time is 7 days, the average ultimate bearing capacity increases to 79.33 kN, which is 84.6% of the maximum ultimate bearing capacity. Generally, the setting time in real operation is 2 days; hence, the reduction factor of the ultimate bearing capacity takes 0.679.

**Figure 8:**
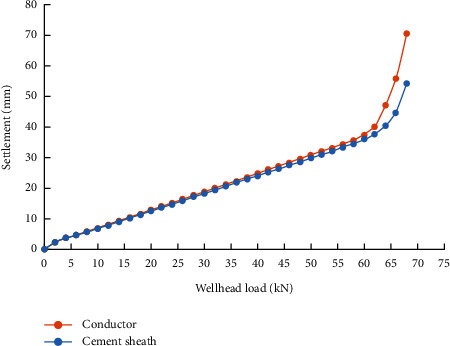
Comparison of settlement between the conductor and cement sheath when setting time is 1 day.

**Figure 9:**
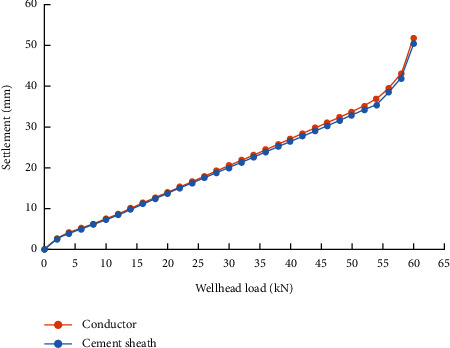
Comparison of settlement between the conductor and cement sheath when setting time is 2 days.

Comparing the Q-z curve of the cement sheath and of the conductor when the setting time is shorter than 2 days (Figure 8), it is found that the settlement of conductor is greater than that of cement sheath under the same load. This indicates that the first cemented surface between the conductor and the cement sheath is damaged at this time and that the soil can no longer provide bearing capacity through the cement sheath. However, when the setting time exceeds 2 days, the Q-z curve of cement sheath coincides with the Q-z curve of the conductor, indicating that the conductor and the cement sheath are completely consolidated into a whole and support the weight of the wellhead equipment when the setting time exceeds 2 days (Figure 9).

## 5. Conclusions

1. A calculation model of axial bearing capacity of the conductor with the size of the conductor and the return height of cement slurry as variables was established on the basis of the hole enlargement that often occurs during drilling. Compared with a series of simulation experimental data of ultimate bearing capacity of conductor, it is concluded that the accuracy of the calculation model is more than 95%.
2. The experimental results showed that the ultimate bearing capacity of the conductor increased first and then tended to be stable with the increase of the setting time. When the setting time is 2 days, the average value of the ultimate bearing capacity increases to 63.67 kN, which is 67.9% of the maximum ultimate bearing capacity. Generally, the setting time in real operation is 2 days; hence, the reduction factor of the ultimate bearing capacity takes 0.679.
3. Comparing the Q-z curve of the cement sheath and of the conductor when the setting time is shorter than 2 days, it is found that the settlement of conductor is greater than that of cement sheath under the same load. This phenomenon indicates that the conductor and the cement sheath are completely consolidated into a whole and support the weight of the wellhead equipment when the setting time exceeds 2 days.

## Figures and Tables

**Table 1:** Soil parameters.

No.	Feature description	Top depth (m)	Bottom depth (m)	Lateral friction (kPa)	Tip resistance strength (MPa)
1	Clay	0	2.1	13	0.38
2	Silty clay	2.1	4.9	21	1.46
3	Silty clay	4.9	6.1	26	3.56
4	Silty clay	6.1	6.5	18	1.20
5	Silty clay	6.5	7.6	13	0.90
6	Silty clay	7.6	8.5	34	2.61
7	Silty clay	8.5	13.1	19	1.30

**Table 2:** Ultimate bearing capacity of conductors under different cement sheath return heights.

No.	Cement sheath return height/m	Ultimate bearing capacity/kN		Error rate (%)	Reduction rate
		Calculated value	Measured value		
1	0	93.8	98	4.47	--
2	−0.5	90.0	94	4.44	4.08%
3	−1.0	86.2	90	4.41	8.16%
4	−1.5	82.4	84	1.94	14.28%
5	−2.0	78.7	80	1.65	18.37%
6	−2.5	74.2	76	2.43	22.45%
7	−3.0	69.5	72	3.59	26.53%
8	−3.5	64.9	68	4.78	30.61%

**Table 3:** Ultimate bearing capacity of conductor under different setting times.

No.	Setting time/d	Measured value of ultimate bearing capacity/kN		
		Group 1	Group 2	Group 3
1	0.5	50	46	48
2	1	56	54	52
3	2	62	62	64
4	3	68	72	66
5	4	74	76	78
6	7	80	78	80
7	14	84	86	84
8	28	88	90	88

## Data Availability

The data used to support the findings of this study are available from the corresponding author upon request.

## Nomenclature

*F*: Bearing capacity provided by the conductors, kN
*F*_*f*_: Lateral friction of formation to cement sheath of conductors, kN
*F*_*q*_: Tip resistance force of assembly of conductors and cement sheath, kN
*W*_*c*_: Total weight of conductors under actual operating conditions, kN
*F*_*i*_: Lateral friction provided by the ith layer, kN
*f*_*i*_: Unit lateral friction of the ith soil layer, kPa
*d*: The actual size of the drilling hole
*d*_*c*_: Diameter of utilized bit, *m*
*H*_*i*_: Thickness of the ith soil layer, *m*
*f*_*n*_: Unit lateral friction of the nth soil layer, kPa
*L*: Penetration depth of conductor under mudline, *m*
*q*_*n*_: Tip resistance strength provided by soil layer, MPa
*A*_*n*_: The cross-sectional area (CSA) of the cement sheath, m2
*m*_*c*_: Line weight of the conductor, kg/m
*x*: Distance from cement sheath to cement top, *m*
*λ*: Volume shrinkage of cement slurry after consolidation
*W*_*h*_: Wellhead load, kN
*W*_*1*_: Wellhead equipment weight, kN
*W*_*2*_: Technical casing weight sits at the wellhead, kN
*n*: Number of soil layers contacted with cement sheath
*β*: Borehole diameter expansion coefficient
*r*_*i*_: Bit radius, *m*; r-radius at wellbore collapse, *m*
*η*: Nonlinear correction coefficient of stress
*p*: Transition variable of *σ*h1, *σ*h1 and *β*
*C*: Cohesion, kN
*K*: Transition variable of Φ
*α*: Effective stress coefficient
*p*_*p*_: Formation pore pressure, kPa
*Q*: Transition variable of *σ*h1, *σ*h1 and *β*
*ρ*_*m*_: Density of cement slurry, kg/m3
*H*: Distance from wellhead to cement top, *m*
*σ*_*h1*_: Maximum horizontal stress, kPa
*σ*_*h2*_: Minimum horizontal in-situ stress, kPa
*Φ*: Angle of internal friction.
